# Adulteration of Iodine

**Published:** 1846-06

**Authors:** M. Herberger


*Adulteration of Iodine.* By M. Herberger.—The present high price of iodine of course leads to its being adulterated. Sulphuret of antimony and carburet of iron are used for this purpose to a considerable extent. To detect these adulterations, the iodine should be sublimed at a gentle heat; the sophistications will of course be left.

